# Spatial inhibition of return as a function of fixation history, task, and spatial references

**DOI:** 10.3758/s13414-016-1123-6

**Published:** 2016-05-13

**Authors:** Jasper H. Fabius, Martijn J. Schut, Stefan Van der Stigchel

**Affiliations:** Experimental Psychology, Helmholtz Institute, Utrecht University, Heidelberglaan 1, 3584 CS Utrecht, The Netherlands

**Keywords:** Inhibition of return, Visual search, Spatial localization

## Abstract

In oculomotor selection, each saccade is thought to be automatically biased toward uninspected locations, inhibiting the inefficient behavior of repeatedly refixating the same objects. This automatic bias is related to inhibition of return (IOR). Although IOR seems an appealing property that increases efficiency in visual search, such a mechanism would not be efficient in other tasks. Indeed, evidence for additional, more flexible control over refixations has been provided. Here, we investigated whether task demands implicitly affect the rate of refixations. We measured the probability of refixations after series of six binary saccadic decisions under two conditions: visual search and free viewing. The rate of refixations seems influenced by two effects. One effect is related to the rate of intervening fixations, specifically, more refixations were observed with more intervening fixations. In addition, we observed an effect of task set, with fewer refixations in visual search than in free viewing. Importantly, the history-related effect was more pronounced when sufficient spatial references were provided, suggesting that this effect is dependent on spatiotopic encoding of previously fixated locations. This known history-related bias in gaze direction is not the primary influence on the refixation rate. Instead, multiple factors, such as task set and spatial references, assert strong influences as well.

**Electronic supplementary material:**

The online version of this article (doi:10.3758/s13414-016-1123-6) contains supplementary material, which is available to authorized users.

Humans and other animals sample their environment with high spatial resolution by fixating different objects for short amounts of time. However, only a single location can be fixated at a time. Since the seminal work of Yarbus (1967), it is known that not all parts of the visual world are fixated equally often. Hence, to efficiently sample the environment for visual information, humans continuously make decisions about where to move their eyes next. These saccadic decisions are influenced by a wide range of factors, such as the stimulus properties, task set, and expectations (for an overview, see Hayhoe & Ballard, 2011). In this context, refixations (fixating an already fixated location or object) have gained considerable attention. The interest in refixations has particularly grown since the first reports of inhibition of return (IOR; Posner & Cohen, 1984; Posner, Rafal, Choate, & Vaughan, 1985). IOR is a delay in responses to recently attended locations at late cue–target onset asynchronies. It has been hypothesized that this temporal delay is enabled by the automatic placement of “inhibitory tags” at previously fixated locations (Abrams & Dobkin, 1994; Klein, 1988; Klein & Macinnes, 1999), thereby lowering the probability of making a refixation, and increasing sampling efficiency (Klein, 2000).

Despite the established temporal effect of IOR, a lowered probability of refixating any given location is often merely inferred from latency data. The increased latencies (temporal IOR) have been hypothesized to reflect a facilitation of saccades toward uninspected locations (spatial IOR), thus increasing sampling efficiency (Klein, 2000). Only a few studies have directly addressed refixation probabilities (Boot, McCarley, Kramer, & Peterson, 2004; Gilchrist & Harvey, 2000; Hooge, Over, van Wezel, & Frens, 2005; Luke, Smith, Schmidt, & Henderson, 2014; McCarley, Wang, Kramer, Irwin, & Peterson, 2003; Smith & Henderson, 2011). Unfortunately, what to use as a baseline when addressing refixation probabilities has been the subject of some debate. As was illustrated by Yarbus’s work, some locations in a scene have a higher probability of being fixated, and therefore subsequently refixated. Hence, when addressing refixation probabilities, multiple parameters (e.g., saliency) have to be controlled for in the baseline probability (Bays & Husain, 2012; Gilchrist & Harvey, 2000; Hooge et al., 2005; Klein & Hilchey, 2011; Smith & Henderson, 2011). McCarley and colleagues (2003) circumvented a complex model with multiple parameters by using an artificial search task consisting of a series of binary saccadic decisions. Their subjects were presented a “hidden search display” where only two items of the entire search array were visible. Subjects made a saccade to either of the two, in order to identify it as a target or a distractor. At some point in the trial, one of the two items was an item that had already been fixated. Hence, the a priori chances of a refixation and of a saccade to a new location were both .5. The results showed that the probability of making a refixation was indeed reduced, but this probability increased to baseline chance with more intermittent fixations, suggesting a limited lifetime of the inhibitory tags. This observation resulted in the hypothesis that IOR tags are stored in visual working memory (VWM; Bays & Husain, 2012; Henderson & Hollingworth, 1999; Hollingworth & Luck, 2009; Peterson, Kramer, Wang, Irwin, & McCarley, 2001). The logic is that because the capacity of VWM is limited (Luck, 2008), information obtained at previous fixations is only available for a limited time. When the information is no longer available in VWM, a saccade might be executed to the location containing the interesting information again.

However, as was noted by Posner et al. (1985), IOR is “not the main determiner,” but rather just one of the many factors contributing to oculomotor behavior. Smith and Henderson (2011) also provided an integrative explanation of refixations, in which IOR is implemented as an initial delay in return saccades, that in more complex tasks might be obscured by other processes. This implies that in certain conditions the suppressing effect of IOR on refixations is stronger than in others. Interestingly, examples in the literature have suggested that the expression of temporal IOR is also modulated by different factors. For example, temporal IOR is observed most strongly when a subject performs a search task, but also to a lesser extent when the subject performs a memory task or is asked to rate a scene for its pleasantness (Dodd, Van der Stigchel, & Hollingworth, 2009). Another example is that temporal IOR diminishes when targets reliably appear at a particular, previously fixated location (Farrell, Ludwig, Ellis, & Gilchrist, 2010). Some flexibility in the rate of refixations has also been observed in the aforementioned binary saccadic decision paradigm (Boot et al., 2004). When subjects were explicitly instructed to intentionally make saccades to new targets instead of refixations, subjects made fewer refixations. This led to the conclusion that the rate of refixations can intentionally be altered. However, whether any flexibility in the rate of refixations is also implicitly influenced by task set has not yet been addressed. On a more global scale, previous studies have suggested that gaze direction is influenced by the current behavioral goals of the observer (Tatler, Wade, Kwan, Findlay, & Velichkovsky, 2010).

Here, we tested the hypothesis that refixations are flexibly inhibited when this is beneficial for task performance, but to a lesser extent when there is no explicit gain from inhibiting refixations. In other words, do fewer refixations occur when task performance profits from inhibiting them, than under neutral, free viewing conditions? To address this question, we used a paradigm similar to that of McCarley et al. (2003). We manipulated the relevance of applying inhibition of refixations by having subjects perform two tasks within the same paradigm. In one task, subjects searched for a specific target (similar to McCarley et al., 2003), where inhibiting refixations would result in increased task performance. In the second task, subjects made saccades without any secondary objective. In this task, inhibiting refixations would not increase task performance.

## Experiment 1

In Experiment 1, we assessed whether differences in the task set result in different saccadic decisions. Subjects completed two versions of a task in which they made six successive saccadic decisions. The decisions were binary (“fixate location A or location B”). The first five decisions were always between two locations that had not been fixated before. Crucially, the final decision was between a location that had been fixated and a novel location. To test the hypothesis that inhibiting refixations is task-dependent, subjects performed the task twice, once when they were instructed to locate a target (“search”), and once when they were instructed to make a series of saccades until a trial ended (“free viewing”). Additionally, we tested whether the probability of refixations increased with more intermittent fixations, since this had previously been observed in a similar search task (McCarley et al., 2003). For our paradigm, this meant that at the final saccadic decision, subjects had to choose between a novel location and a location that had been fixated either one, two, three, or four fixations back.

### Method

#### Subjects

Ten naïve subjects (ages 20–27; nine female, one male) with normal or corrected-to-normal vision participated in Experiment 1. All subjects gave informed written consent and were paid for their participation. The study was approved by the faculty ethics committee of Utrecht University and was conducted in accordance with the Declaration of Helsinki.

#### Apparatus

All stimuli were presented on an LG 24MB65PM LCD-IPS monitor (50.7 × 33.9 cm) with a spatial resolution of 1,280 × 800 and a refresh rate of 60 Hz. The stimuli were generated using MATLAB (The MathWorks Inc., Natick, MA) and the Psychophysics Toolbox 3.0 (Brainard, 1997; Pelli, 1997). Eye movements were recorded with an EyeLink 1000 eyetracker (SR Research Ltd., Ottawa ON) with a sampling rate of 1000 Hz. The left eye was monitored. Subjects were seated in a darkened room and viewed the screen from a distance of 70 cm; their heads rested on a chinrest with a forehead rest, to minimize movements.

#### Stimuli

**Fig. 1:**
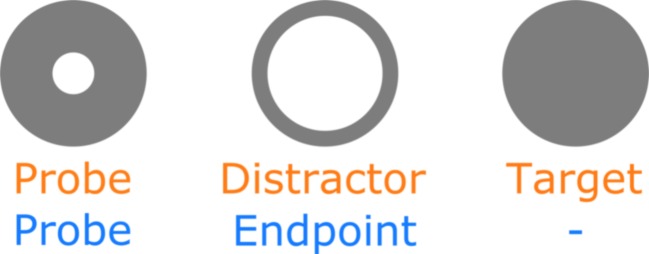
Probes, distractor, and target. In both the search task (top labels) and the free viewing task (bottom labels), the probes were used as potential saccade targets. The actual diameter was 0.5°. The probe changed into either a distractor or a target in the search task. In the free viewing task, the probes did not change upon fixation. Only the final probe in each trial changed into the “endpoint,” which was similar to a distractor in the search task

Locations were probed by small, thick gray rings (radius = 0.25°, radius inner circle = 0.14°; see Fig. 1), presented on a black background. In the search task, upon fixation the probes changed into thin gray rings, indicating a distractor (radius = 0.25°, radius inner circle = 0.2°), or a filled gray circle, indicating the target (radius = 0.25°). In the free-viewing probes, only the final probe changed from the thick gray ring to a thin gray ring, indicating the trial end. The corners of the area where the probes could appear were marked by differently colored (red, green, yellow, and blue), orthogonal lines (2°), similar to those used in McCarley et al. (2003).

#### Procedure

**Fig. 2:**
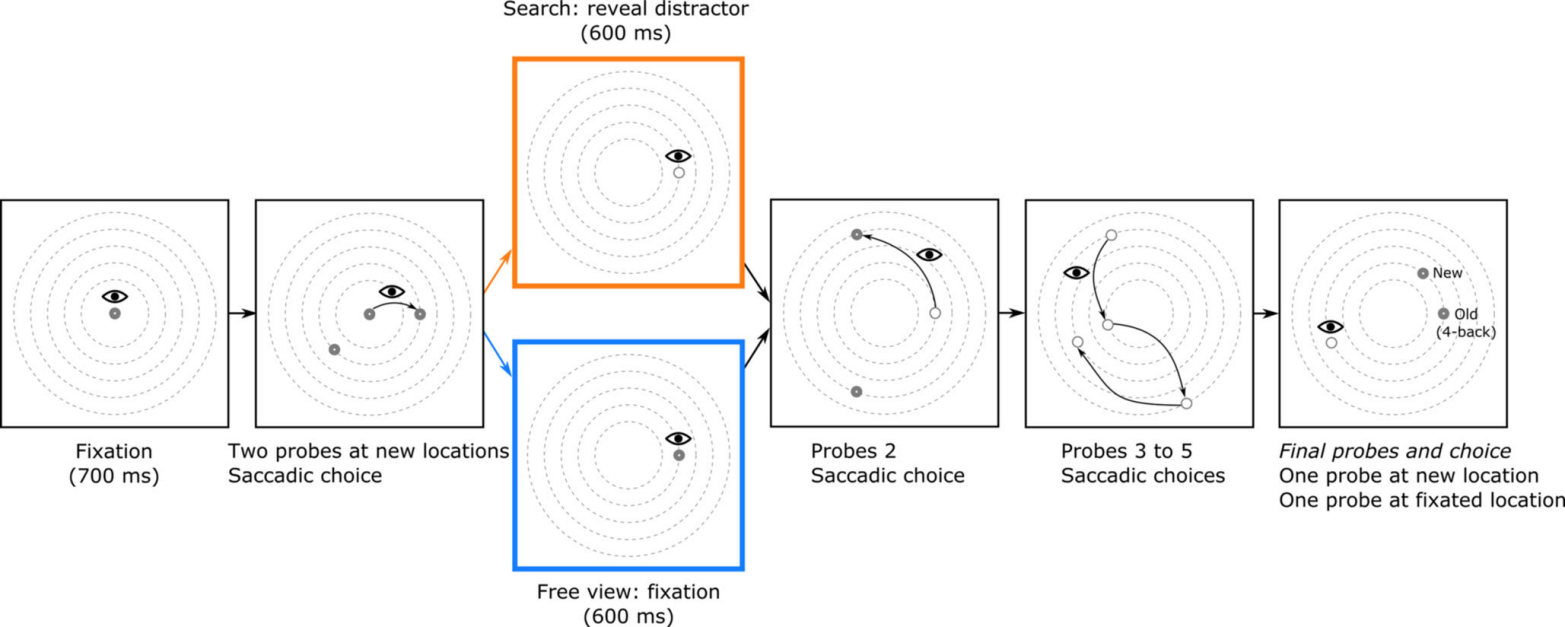
Sequence of events in a typical experimental trial with a lag of 4. In the actual display, the probe size was twice as small with respect to the dotted eccentricity rings depicted here. The actual background color was black, and the dotted rings were not visible. (1) A trial started with a central fixation for 700 ms, followed by the appearance of the first two location probes. (2) Subjects were instructed to make a saccade to either probe. (3) In the search task, the fixated probe turned into a distractor. In the free-viewing task, the probe did not change. There was always a delay of 600 ms between fixation and the onset of the two subsequent location probes. (4) Another saccadic decision was made. (5) For illustration purposes, the alternative probe locations for Saccades 3–5 are left out, so that only the fixated locations are shown. In the actual experiments, subjects were presented two location probes at each step. Note that the distractors displayed here would not remain on screen. Moreover, in the free viewing task, the probes never changed into a distractor until the final probe. (6) At the final screen, one probe was located at a position that had been fixated before, and one at an uninspected location

Subjects completed two tasks: “search” and “free viewing.” The order of the tasks was counterbalanced across subjects. Both consisted of 240 experimental trials. The search task had an additional 240 filler trials randomly interleaved with the experimental trials (explained below). The search task was divided into eight blocks, the free viewing task into four. All blocks started with the standard 9-point calibration and validation routines of the EyeLink 1000 eyetracker. In the experimental trials (Fig. 2), subjects initially fixated a central fixation point. After 500 ms of stable fixation, two location probes appeared. Subjects were instructed to fixate one of the two probes. The alternative and the previously fixated probe (or fixation point) disappeared upon the new fixation. After 600 ms, two new probes were presented, and the subject made another saccadic decision and fixated one of the two probes. Subjects made six decisions per trial, in which each new probe pair was shown 600 ms after fixation onset. This interval was fixed. In the first five pairs, both probes were located at novel locations. In the final pair, one probe was located at a novel location, and the other probe was located at one of the previously fixated locations. The “old” location could be either one, two, three, or four fixations back. There were 60 trials for each of these lags. The choice in this final saccadic decision was used as a measure of saccadic choice preference.

The search task and free viewing task differed in their instructions. In the search task, subjects were instructed to locate a target stimulus. In the free viewing task, they were instructed to fixate one probe of each pair until the trial ended. In addition to the difference in instructions, the experimental trials differed slightly between the different tasks. In the search task, a fixated probe “revealed its identity” upon fixation—that is, changed into a distractor (thin ring) or a target (filled circle). Importantly, this target was always located at the new location in the final probes. Thus, subjects would only find the target when they did not make a refixation. In the free viewing task, the location probes did not change, but remained thick gray rings. Only the last probe changed into a thin gray ring upon fixation, to indicate the trial end. However, in this task it did not matter whether a subject made a refixation; the trial would end, regardless. In both tasks, the alternative and previous probes disappeared from the screen upon a new fixation. Note that despite small differences in the foveal stimuli between the two tasks, peripheral visual stimulation during the crucial part of saccadic decision making was equal in both tasks.

In addition to the experimental trials, the search task contained 240 filler trials. In these filler trials, subjects would always find the target at either the first (20%), second (20%), third (20%), fourth (20%), or fifth (20%) fixation, irrespective of which location of a probe pair was fixated. All probes in the filler trials were shown at uninspected locations, similar to the first five probe pairs of the experimental trials. The filler trials were included to keep the subjects actively involved in their saccadic decisions, by giving the impression that the location of the target was really predetermined, whereas it was actually determined gaze-contingently. In other words, the location of the target was not set at the beginning of a trial, but rather the time at which it would be shown was set (i.e., always after the sixth decision in experimental trials, and always before the sixth decision in filler trials).

#### Probe locations

Locations were probed gaze-contingently, to ensure that two probes were placed equidistant from the currently fixated location. Locations were set in polar coordinates, using a set of five fixed eccentricities (*ρ*) with respect to the center of the screen (depicted as the dotted rings in Fig. 2). The *ρ*s were 3°, 4.5°, 6°, 7.5°, and 9° of visual angle. The sequence of *ρ*s was shuffled, with the constraint that for two consecutive probe pairs, the *ρ*s differed by at least 3° of visual angle. The angular separation (*θ*) between the first two probes was 120 deg. For the next probe pairs, the angular separation was 90 deg when *ρ* increased. When *ρ* decreased, the two probes were placed on opposing sides of the imaginary circle around screen center with a radius *ρ* (so, the distance between the two probes was 2*ρ*). These constraints yielded a median separation of 9.0° between the two probes in a pair (min 4.1°, max 18.0°) and a median distance of 8.7° between the currently fixated probe and the next probes (min 2.3°, max 16.3°). To anticipate and prevent situations in which it would have been impossible to pick two locations meeting these constraints, all possible sequences for every trial (i.e., 2^6^ sequences) were computed prior to the experiment.

#### Data analysis

Online gaze analysis was based on eye position. Targets were revealed when gaze was detected within a region of 2° around either probe. The saccades and fixations were reanalyzed offline with a velocity-based algorithm (Nyström & Holmqvist, 2010). Trials were excluded when saccades after the presentation of the final probes were either too fast (<80 ms) or too slow (>1,000 ms). Second, trials were excluded when no fixations were detected after the onset of the final probes or when the final fixation was not decisively close to one of the two probes (0.8%–7.9%). A third exclusion criterion was when the online gaze-contingent algorithm failed to detect gaze samples at either probe within 2,600 ms after probe onset (1.0%–6.9% of trials per subject). These exclusion criteria resulted in a minimum of 45 trials per lag per subject in each task.

We performed a logit mixed-effects analysis using the lme4 package in R (Baayen, Davidson, & Bates, 2008; Bates, Mächler, Bolker, & Walker, 2015; Jaeger, 2008). In this model we included task and lag as fixed effects, and for each subject a random intercept. “Lag 1” in the search task was set as the reference level. With these settings, all reported *β*s (in log probability) are relative to the rate of refixations at lag 1 in the search conditions.

### Results

#### Refixation rate

**Fig. 3:**
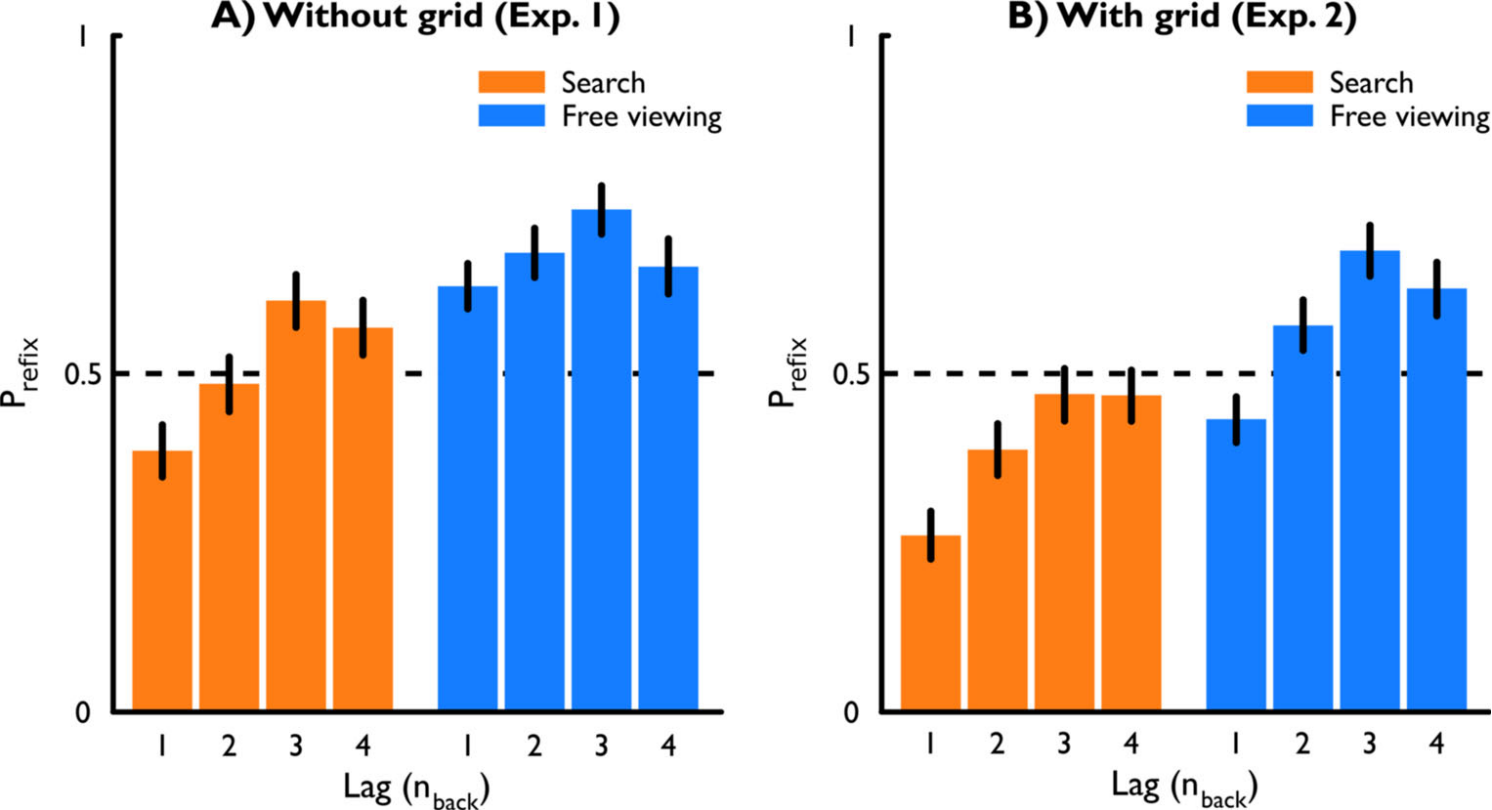
Proportions of refixations. (A) Experiment 1: Subjects performed the tasks on a blank background. (B) Experiment 2: Subjects performed the task with a continuously present background grid. In both panels, the left bars represent the group average proportions of refixations in the search task, and the right bars represent the group average proportions of refixations in the free viewing task. Error bars represent the bootstrapped 95% confidence intervals of the sample mean (2,000 bootstrap samples). Values smaller than .5 indicate a preference for new locations, and values above .5 indicate a preference for refixations

Figure 3 (left panel) shows the proportions of refixations at different lags in both tasks: search and free viewing. As can be seen in the figure, the probability of refixations seems to increase until a lag of 4. After visual inspection of the data, we analyzed a linear effect of lag (in log space) from lag 1 to lag 3. Including the data from four-back would reduce the fit of the model, or would require an overparameterized, nonlinear model. We believe it is fair to assume that from lag 3 onward, a constant “plateau” in the rate of refixations is reached, and that any fluctuations there are related to noise rather than a fixed effect.

We found a preference for saccades toward new targets in the search task at lag 1 (*β* = –0.52, *z* = 3.64, *p* < .001). However, there were more refixations with increasing lag in the search task (*β* = 0.48, *z* = 7.66, *p* < .001). In the free viewing task, the rate of refixations at lag 1 was considerably higher than in the search task (*β* = 1.06, *z* = 9.212, *p* < .001). To inspect the free-viewing condition further, we reran the model with this task as a reference level. At lag 1, subjects showed a preference for refixations in the free viewing task (*β* = 0.55, *z* = 3.79, *p* < .001), in contrast to the preference for saccades to new targets in the search task. Moreover, although an effect of lag did emerge in the free viewing task (*β* = 0.27, *z* = 4.02, *p* < .001), it was substantially smaller than in the search task (*β* = 0.21, *z* = 2.29, *p* = .022).

On the basis of bootstrapped 95% confidence intervals (see Supplementary Table S1), we observed significant “absolute” inhibition of return only in the search task at lag 1. In contrast, we observed a preference for refixations at lags 3 and 4 in the search task and for all lags in the free viewing task. In summary, Experiment 1 shows a preference for refixations in the free viewing task that grows stronger with increasing lag. Furthermore, in the search task, in which refixations decreased task performance, there was a preference for saccades toward new locations at the shortest lag, but this increased with increasing lag. The refixation rate increased even to the extent that a preference for refixations was observed for lags 3 and 4.

#### Saccade latency

Although subjects were not instructed to make saccades as quickly as possible, but rather to find as many targets as possible (in the search task), we analyzed the saccadic latencies (see Supplementary Table S2 with all of the mean latencies). This analysis was performed because IOR is often defined as an increased latency between onset and response. Since we had no clear baseline latency, we included Choice (refixation vs. new) as a factor, so that the linear mixed-effects analysis on the saccadic latencies included Choice, Lag, and Task as fixed factors and subjects as an effect on the intercept. The reported *β*s are in milliseconds, with respect to the average latency for saccades toward new locations in the search task. Statistics are reported with *t* values only. As a rough approximation, *t* values higher than 2 are usually considered as a significant difference (Baayen et al., 2008).

The estimated latency for saccades toward new locations in the search task at lag 1 was 228.8 ms. This was not significantly different for saccades toward already fixated locations (*β* = –0.14, *t* = 0.019). Latencies in the free-viewing task were not significantly higher than in the search task (*β* = 0.4, *t* = 0.05), nor was the latency difference between saccades toward already fixated and new locations more pronounced (*β* = 6.0, *t* = 0.56). We found no effect of lag in either the search task (*β* = 4.5, *t* = 1.14) or the free-viewing task (*β* = –6.7, *t* = 1.054). In the search task, the effect of choice (refixation vs. new location) was not significantly different for shorter than for longer lags (*β* = –8.3, *t* = 1.47), which was also not different in the free viewing task (*β* = 2.8, *t* = 0.34). To summarize, neither choice, lag, nor task was a significant predictor of saccadic latencies (for a full overview of the estimated parameters and *t* statistics, see Supplementary Table S3). This presumably implies that factors other than the classic IOR effect more strongly affected latencies (for a similar notion, see Smith & Henderson, 2011). It should be noted that since subjects were not instructed to make speeded saccades, any subtle effect might have been obscured.

### Discussion

In Experiment 1, we used a binary saccadic decision paradigm to quantify saccadic choice preferences for new locations and refixations under two different task sets. A similar paradigm has been used to show that in visual search, saccades toward new locations are favored over refixations (McCarley et al., 2003). This observation has been linked to the phenomenon of IOR (Macinnes & Klein, 2003), in which saccades to probes have been found to be slower when directed to probes presented at recently fixated locations. However, subsequent studies have shown that this temporal slowing of refixations is specific to visual search, and is not observed (or only to a lesser extent) in other visual tasks (Dodd et al., 2009; Smith & Henderson, 2009).

In Experiment 1, we showed that saccadic decisions are mediated by both a history-related effect and a task-related effect. With increasing lag between the initial fixation and the final decision, there was a higher rate of refixations. In addition to an effect of lag, we also observed an effect of task on the rate of refixations, with more refixations in the free viewing task as compared to the search task. Interestingly, we observed *absolute* spatial IOR only for the most recently fixated location and only in the search task. In contrast, in the free viewing task, refixations were favored over saccades to new locations. This suggests that refixations may occur frequently by default under task settings other than search. Moreover, they are actively inhibited during search, but the effect of lag on the rate of refixations is present in both search and free viewing. Hence, this effect might reflect an automatic process, such as IOR (Klein & Macinnes, 1999) or saccadic momentum (Smith & Henderson, 2009), that is intrinsic to the oculomotor system (Hooge & Frens, 2000; Posner et al., 1985).

Despite the similarities in paradigm, there is an important difference between the results of McCarley et al. (2003) and the present experiment: whereas in both experiments a similar lag-related effect was observed, we did not observe the *absolute* spatial IOR that was found in the original paradigm. We believe that small differences between the paradigms may have resulted in this difference. In McCarley et al.’s paradigm, stimuli that had been fixated could remain on screen over the course of several saccades. In contrast, in the present experiment, all stimuli except the fixated stimulus were removed from the screen at the onset of fixation. Therefore, in the present experiment, subjects only had a single opportunity to make a refixation in every trial, whereas in the original paradigm a previously fixated item could reappear several times on screen, or even remain on screen over several saccades. Importantly, this might have facilitated spatiotopic encoding of IOR in the original paradigm (Klein & Macinnes, 1999; Müller & von Mühlenen, 2000; Takeda & Yagi, 2000). Indeed, McCarley and colleagues noted that the rate of refixations was lower when items remained on screen (McCarley et al., 2003) or when more spatial references were provided (Kramer, McCarley, Boot, & Peterson, 2004).

To investigate whether sufficient spatial reference is a prerequisite for successfully inhibiting refixations of previously fixated locations, we performed a second experiment with a different group of subjects (*n* = 10). Experiment 2 was essentially a replication of Experiment 1, with the addition of a radial grid in the background display to provide more spatial reference.

## Experiment 2

**Fig. 4:**
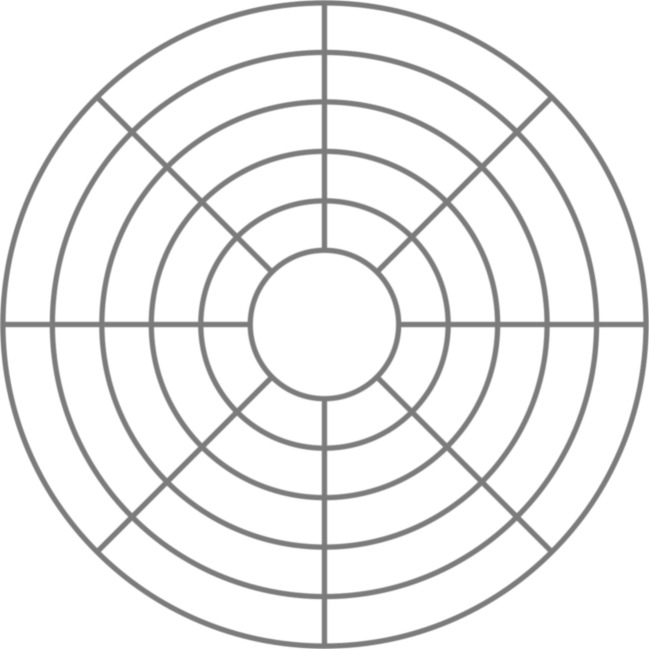
A radial grid was added to the display in Experiment 2. Targets could only appear between the concentric lines

In Experiment 2, we investigated the hypothesis that with continuous spatial references, the rate of refixations can be reduced. Both tasks from Experiment 1 were repeated with a different set of subjects and the addition of a radial grid (Fig. 4) in the display, to facilitate the spatiotopic encoding of previously fixated locations.

### Method

Ten different naïve subjects (ages 19–26; nine female, one male) participated in Experiment 2. All apparatus, stimuli, and procedures were identical to those of Experiment 1, with the addition of a radial grid (Fig. 4) to the background of the display. This grid was present during an entire trial, and the location probes appeared between the radial lines of the grid. The same exclusion criteria as in Experiment 1 were used, resulting in at least 46 trials per lag per condition.

### Results

#### Refixation rate

Figure 3 (right panel) shows the logits of refixations at different lags for the two conditions obtained in Experiment 2. To investigate whether the addition of a radial grid to the display decreased the rate of refixations, we performed another linear mixed logit effects analysis. We included the factors Task, Lag, and Grid Presence as fixed effects, and subject as a random effect. The reference level for all subsequently reported *β*s is the search task in Experiment 1 (without grid) at lag 1. As we found before, there was a significant preference for saccades toward new locations in the search task at lag 1 (*β* = –0.52, *z* = 3.95, *p* < .001). In Experiment 2, this preference was even more pronounced (*β* = –0.49, *z* = 2.59, *p* = .009). The effect of lag in Experiment 1 had been expressed as an increasing preference for refixations with increasing lag (*β* = 0.48, *z* = 7.65, *p* < .001). In Experiment 2, a similar effect was observed, not significantly different from the effect of lag in Experiment 1 (*β* = 0.003, *z* = 0.031, *p* = .975). Furthermore, in Experiment 1 the refixation rate was higher in the free viewing task than in the search task (*β* = 1.06, *z* = 9.21, *p* < .001); in Experiment 2, this difference was slightly smaller (*β* = –0.33, *z* = 2.00, *p* = .045), although there was still a higher rate of refixations in the free viewing task than in the search task (*β* = 0.73, *z* = 6.25, *p* < .001). The effect of lag was smaller in the free viewing task than in the search task in Experiment 1 (*β* = –0.21, *z* = 2.29, *p* = .022). However, in Experiment 2, the effect of lag was not different across the different tasks (*β* = 0.06, *z* = 0.64, *p* = .525). In summary, we observed a reduction in refixations in both the search and free viewing tasks when a background grid was present. Moreover, there was an effect of lag irrespective of grid presence, yet this effect was stronger in the free viewing task when a background grid was provided. This difference was not observed in the search task.

As in the analysis of Experiment 1, we further inspected the observed refixation rates with bootstrapped 95% confidence intervals (see Supplementary Table S1). When a background grid was present, a preference for saccades toward new locations was apparent in both the search and the free viewing task at lag 1. In the search task, this was also the case at lag 2, but in the free viewing task, there was a preference for refixations at lag 2. This preference for refixations was also found for lags 3 and 4 in the free viewing task. In the search task, however, no clear preference for either probe emerged at lags 3 and 4.

#### Saccade latency

As in Experiment 1, although we did not instruct subjects to finish a trial as quickly as possible, we analyzed saccadic latencies, since they are such an important measure in the IOR literature (see Supplementary Table S2). We used a linear mixed-effects analysis with Choice, Task, Lag, and Grid Presence as fixed factors and subject as a random effect. The output of this analysis is provided in Supplementary Table S4. In short, this analysis showed that saccadic latencies in the search task increased with the introduction of a background grid (*β* = 10.8, *t* = 4.36), and that this increase was smaller in the free viewing task (*β* = –5.4, *t* = 4.05). We observed no lag- or refixation-related effects (all *t*s < 1.2). The increased latency in the search task suggests that subjects might have employed more cognitive strategies to prevent refixations in the search task.

### Discussion

To facilitate spatiotopic encoding of previously fixated locations, a radial grid was added to the background. This background grid was not relevant to the task in any way, but simply provided more spatial references to the display than in Experiment 1. We observed that the rates of refixations were reduced when sufficient spatial references were provided. In regular search displays, these references can comprise the items in the search display itself.

The data show that the reduction in refixations as a result of the background grid was not task-specific. Importantly, with a background grid, we observed a quantitative preference for saccades toward new locations (i.e., spatial IOR) in the search task up to lag 2, and in the free viewing task at lag 1. However, there was still a preference for refixations in the free viewing task from lag 2 onward.

We believe that the presence of continuous visual stimuli (as in Exp. 2) may account for the differences in absolute refixation rates between Experiment 1 and previous experiments (Boot et al., 2004; McCarley et al., 2003). In the previous experiments, stimuli could be present on the screen for several fixations, in contrast to the present study, in which stimuli always disappeared upon the next fixations. Instead of adding persistent probes to the display, we decided to use a background grid instead, to keep most parameters constant from Experiments 1 to 2, enabling a fairer comparison between the two.

## General discussion

Biases in saccadic decisions have been found to favor saccades toward uninspected locations, at least during visual search (Gilchrist & Harvey, 2000; McCarley et al., 2003; Peterson et al., 2001). This bias has been hypothesized to result from an automatic process (Boot et al., 2004) such as IOR (Klein & Macinnes, 1999; Macinnes & Klein, 2003). IOR is commonly defined in the temporal domain as an increase in the latencies of responses to recently attended stimuli (Posner & Cohen, 1984). This increase in latencies has been suggested to facilitate visual search by decreasing the probability of making a refixation (Klein, 1988, 2000). Studies have indicated flexibility in the expression of temporal IOR under task sets other than visual search (Dodd et al., 2009; Farrell et al., 2010; Luke et al., 2014). Moreover, it has been stressed that the process of making a refixation is subject to multiple factors (Posner et al., 1985; Smith & Henderson, 2011), of which at least one can be flexibly adjusted. This has been interpreted to reflect an efficient flexibility to adapt oculomotor behavior to meet the current task demands. Here we tested this functional interpretation of IOR by having subjects perform a nearly identical paradigm under two different sets of instructions.

The present data show both a task-dependent and a history-dependent effect on the rates of refixations. Moreover, refixation rates were lower when more spatial references were provided. It has been noted before that the refixation rate is inflexibly influenced by an automatic, history-dependent process (Boot et al., 2004; Gilchrist & Harvey, 2000; McCarley et al., 2003; Peterson et al., 2001). This process biases saccades in favor of new locations, suggestively corresponding with the conceptualization of IOR (Klein & Macinnes, 1999; Posner et al., 1985) or saccadic momentum (Smith & Henderson, 2009). Although the history-dependent effect in the present experiments was also present under both task sets, the strength of the effect interacted with task set. Even more, we observed a three-way interaction of task, lag, and spatial references, suggesting that a history-dependent effect can be modulated by task set, but that this modulation is weaker when sufficient spatial references are provided. Thus, speculatively, the influence of the history-dependent effect was stronger with sufficient spatial references, fitting with converging evidence that IOR is coded in spatiotopic coordinates (Wang & Klein, 2010).

As a crucial addition to this history-dependent process, a more flexible process has been suggested to influence saccadic decision as well (Boot et al., 2004; Luke et al., 2014; Smith & Henderson, 2011). Here, we confirmed such a second process, which is implicitly influenced by task demands. When subjects were searching for a target, they made fewer refixations than when they made saccadic decisions without specific search instructions. Moreover, the present data suggest that under specific conditions, refixating might actually be a default mode, even though immediate refixations tend to be inhibited through the aforementioned automatic process (i.e., the high probability of refixations at late lags in free viewing). Under natural viewing conditions, these locations may comprise the most salient regions within a scene (Bays & Husain, 2012; Wilming, Harst, Schmidt, & König, 2013). Moreover, the implicit benefit of inhibiting refixations in a search task only goes for static displays. When targets are mobile, reinspecting a location might be fruitful.

For the oculomotor system to take previously fixated locations into account, those locations should have references in a spatiotopic map (Gabay, Pertzov, & Cohen, 2013; Hilchey, Klein, Satel, & Wang, 2012; Mathôt & Theeuwes, 2010; Posner & Cohen, 1984). It has been suggested that these locations are stored in working memory (Bays & Husain, 2012; Peterson et al., 2001; Shen, McIntosh, & Ryan, 2014). In the artificial search paradigm used here, the maintenance of these locations in working memory was particularly difficult, as all stimuli were removed from the screen when they were no longer fixated. This might be a substantial difference from the similar paradigms that have been used previously, in which stimuli could remain on screen, providing continuous spatial reference (Boot et al., 2004; Kramer et al., 2004; McCarley et al., 2003). Indeed, when we provided subjects with more spatial references, they were better at inhibiting refixations, perhaps as a result of improved working memory representations (Deubel, 2004; Golomb, Pulido, Albrecht, Chun, & Mazer, 2010; Lisi, Cavanagh, & Zorzi, 2015).

The present results show that the probability of a refixation is influenced by at least two processes: one history-related process inhibiting immediate refixations, and one flexible process that can be implicitly influenced by task set. Importantly, the expression of at least the history-related effect seems to be related to the degree to which fixated locations can be maintained spatiotopically. Together, these findings confirm the notion of Posner et al. (1985) that, although the oculomotor system may be intrinsically biased to making saccades toward new locations, other factors play a crucial role as well, even to such an extent that the probability of a refixation is higher than chance. Moreover, the observation of absolute spatial IOR is related to the presence of sufficient spatial references.

## Electronic supplementary material

Below is the link to the electronic supplementary material.

ESM 1 (DOC 30 kb)

ESM 2 (DOC 35 kb)

ESM 3 (DOC 30 kb)

ESM 4 (DOC 35 kb)
