# Supplementary material for: Spatial inhibition of return as a function of fixation history, task, and spatial references
Source: Atten Percept Psychophys. 2016 May 13;78:1633–41. doi: 10.3758/s13414-016-1123-6 (PMC4972844; doi:10.3758/s13414-016-1123-6)
Supplement: Supplementary file 1 — (DOC 30 kb) [file 13414_2016_1123_MOESM1_ESM.doc]

# Supplementary Tables

| Table 1. | Bootstrapped 95% confidence intervals of the proportions of re-fixations (2000 bootstrap samples) | | | |
| --- | --- | --- | --- | --- |
| **Task** | | **Lag** | **No grid** | **Grid** |
| Search | | 1 | 0.35 – 0.43 * | 0.23 – 0.30 * |
|  | | 2 | 0.45 – 0.52 | 0.35 – 0.43 * |
|  | | 3 | 0.57 – 0.65 * | 0.43 – 0.51 |
|  | | 4 | 0.53 – 0.61 * | 0.43 – 0.51 |
| Free saccades | | 1 | 0.59 – 0.67 * | 0.40 – 0.47 * |
|  | | 2 | 0.64 – 0.72 * | 0.53 – 0.61 * |
|  | | 3 | 0.71 – 0.78 * | 0.64 – 0.72 * |
|  | | 4 | 0.62 – 0.70 * | 0.59 – 0.66 * |
