# Supplementary material for: Spatial inhibition of return as a function of fixation history, task, and spatial references
Source: Atten Percept Psychophys. 2016 May 13;78:1633–41. doi: 10.3758/s13414-016-1123-6 (PMC4972844; doi:10.3758/s13414-016-1123-6)
Supplement: Supplementary file 2 — (DOC 35 kb) [file 13414_2016_1123_MOESM2_ESM.doc]

| Table 2. | Average saccadic reaction times in milliseconds | | |  |
| --- | --- | --- | --- | --- |
| **Task** | **Lag** | **Choice** | **No grid (s.e.m.)** | **Grid (s.e.m.)** |
| Search | 1 | new | 230.9 (13.5) | 350.2 (36.3) |
|  |  | re-fixation | 238.2 (16.8) | 344.6 (37.5) |
|  | 2 | new | 221.2 (12.2) | 356.2 (35.3) |
|  |  | re-fixation | 218.2 (10.9) | 352.3 (37.4) |
|  | 3 | new | 243.5 (15.6) | 366.6 (43.0) |
|  |  | re-fixation | 230.0 (12.0) | 344.1 (37.1) |
|  | 4 | new | 234.0 (15.8) | 345.3 (33.8) |
|  |  | re-fixation | 219.0 (10.8) | 334.8 (37.2) |
| Free saccades | 1 | new | 251.6 (13.5) | 322.5 (42.7) |
|  |  | re-fixation | 252.2 (22.9) | 292.6 (31.5) |
|  | 2 | new | 239.3 (15.5) | 292.5 (24.3) |
|  |  | re-fixation | 229.6 (8.5) | 282.4 (21.5) |
|  | 3 | new | 226.8 (8.2) | 295.4 (26.1) |
|  |  | re-fixation | 226.1 (11.3) | 288.1 (27.2) |
|  | 4 | new | 239.6 (10.4) | 278.8 (17.8) |
|  |  | re-fixation | 221.1 (7.8) | 276.5 (23.1) |
