# Supplementary material for: Spatial inhibition of return as a function of fixation history, task, and spatial references
Source: Atten Percept Psychophys. 2016 May 13;78:1633–41. doi: 10.3758/s13414-016-1123-6 (PMC4972844; doi:10.3758/s13414-016-1123-6)
Supplement: Supplementary file 3 — (DOC 30 kb) [file 13414_2016_1123_MOESM3_ESM.doc]

| Table 3. | Results of the linear mixed effects analyses of saccadic latencies in Experiment 1 (without grid) | | | |  |
| --- | --- | --- | --- | --- | --- |
| **Parameter** | | **Estimate** | **Std. error** | **t-value** | |
| Intercept | | 228.8 | 9.1 | 24.98 | |
| Re-fixation | | -0.1 | 7.3 | -0.02 | |
| Free viewing | | 0.4 | 7.6 | 0.05 | |
| Lag | | 4.5 | 3.9 | 1.14 | |
| Re-fixation * Free viewing | | 6.0 | 10.6 | 0.56 | |
| Re-fixation * Lag | | -8.3 | 5.6 | -1.47 | |
| Free viewing * Lag | | -6.7 | 6.4 | -1.05 | |
| Re-fixation * Free viewing * Lag | | 2.8 | 8.3 | 0.34 | |
