# Supplementary material for: Spatial inhibition of return as a function of fixation history, task, and spatial references
Source: Atten Percept Psychophys. 2016 May 13;78:1633–41. doi: 10.3758/s13414-016-1123-6 (PMC4972844; doi:10.3758/s13414-016-1123-6)
Supplement: Supplementary file 4 — (DOC 35 kb) [file 13414_2016_1123_MOESM4_ESM.doc]

| Table 4. | Results of the linear mixed effects analyses of saccadic latencies in Experiment 1 (without grid) and 2 (with grid) | | | |  |
| --- | --- | --- | --- | --- | --- |
| **Parameter** | | **Estimate** | **Std. error** | **t-value** | |
| Intercept | | 229.3 | 17.6 | 13.03 | |
| Grid presence | | 10.8 | 24.7 | 4.36 | |
| Re-fixation | | -0.1 | 9.6 | 0.01 | |
| Free viewing | | 0.3 | 10.1 | 0.03 | |
| Lag | | 4.5 | 5.2 | 0.86 | |
| Grid * Re-fixation | | -15.7 | 14.1 | 1.12 | |
| Grid * Free viewing | | -53.6 | 13.2 | 4.05 | |
| Re-fixation * Lag | | 6.1 | 14.0 | 0.43 | |
| Grid * Lag | | 0.7 | 7.0 | 0.10 | |
| Re-fixation * Lag | | -8.3 | 7.4 | 1.12 | |
| Free viewing * Lag | | -6.7 | 8.4 | 0.80 | |
| Grid * Re-fixation * Free viewing | | -10.4 | 20.0 | 0.52 | |
| Grid * Re-fixation * Lag | | -5.2 | 10.6 | 0.49 | |
| Grid * Free viewing * Lag | | -1.4 | 11.2 | 0.13 | |
| Re-fixation * Free viewing * Lag | | 2.8 | 10.9 | 0.26 | |
| Grid * Re-fixation * Free viewing * Lag | | -0.8 | 15.3 | 0.06 | |
